# Peculiarities of the Crystal Structure Evolution of BiFeO_3_–BaTiO_3_ Ceramics across Structural Phase Transitions

**DOI:** 10.3390/nano10040801

**Published:** 2020-04-21

**Authors:** Dmitry V. Karpinsky, Maxim V. Silibin, Sergei V. Trukhanov, Alex V. Trukhanov, Alexander L. Zhaludkevich, Siarhei I. Latushka, Dmitry V. Zhaludkevich, Vladimir A. Khomchenko, Denis O. Alikin, Alexander S. Abramov, Tomasz Maniecki, Waldemar Maniukiewicz, Martin Wolff, Volker Heitmann, Andrei L. Kholkin

**Affiliations:** 1 Institute of Advanced Materials and Technologies, National Research University of Electronic Technology “MIET”, 124498 Moscow, Russia; sil_m@mail.ru; 2 Laboratory of Technology and Physics of Crystals Growth, Scientific-Practical Materials Research Centre of NAS of Belarus, 220072 Minsk, Belarus; truhanov86@mail.ru (A.V.T.); zheludkevich27@gmail.com (A.L.Z.); smer444@mail.ru (S.I.L.); geludkevichdima@mail.ru (D.V.Z.); 3 Scientific and Educational Center “Nanotechnology”, South Ural State University, 454080 Chelyabinsk, Russia; 4 Institute for Bionic Technologies and Engineering, I.M. Sechenov First Moscow State Medical University, 119991 Moscow, Russia; 5 Scientific-Manufacturing Complex “Technological Centre” Shokin Square, House 1, Bld. 7, Zelenograd, 124498 Moscow, Russia; 6 Center for Physics of the University of Coimbra, Department of Physics, University of Coimbra, 3004-516 Coimbra, Portugal; uladzimir@uc.pt; 7 School of Natural Sciences and Mathematics, Ural Federal University, 620026 Ekaterinburg, Russia; denis.alikin@ua.pt (D.O.A.); alexander.abramov@urfu.ru (A.S.A.); kholkin@ua.pt (A.L.K.); 8 Institute of General and Ecological Chemistry, Lodz University of Technology, 90-924 Lodz, Poland; tomasz.maniecki@p.lodz.pl (T.M.); waldemar.maniukiewicz@p.lodz.pl (W.M.); 9 Institute of Materials Research, Helmholtz-Zentrum Geesthacht, Zentrum für Material- und Küstenforschung GmbH, DE-21502 Geesthacht, Germany; martin.wolff@hzg.de (M.W.); volker.heitmann@hzg.de (V.H.); 10 Department of Physics & CICECO—Aveiro Institute of Materials, University of Aveiro, 3810-193 Aveiro, Portugal

**Keywords:** multiferroics, diffraction, crystal structure, phase transitions

## Abstract

Evolution of the crystal structure of ceramics BiFeO_3_–BaTiO_3_ across the morphotropic phase boundary was analyzed using the results of macroscopic measuring techniques such as X-ray diffraction, differential scanning calorimetry, and differential thermal analysis, as well as the data obtained by local scale methods of scanning probe microscopy. The obtained results allowed to specify the concentration and temperature regions of the single phase and phase coexistent regions as well as to clarify a modification of the structural parameters across the rhombohedral–cubic phase boundary. The structural data show unexpected strengthening of structural distortion specific for the rhombohedral phase, which occurs upon dopant concentration and temperature-driven phase transitions to the cubic phase. The obtained results point to the non-monotonous character of the phase evolution, which is specific for metastable phases. The compounds with metastable structural state are characterized by enhanced sensitivity to external stimuli, which significantly expands the perspectives of their particular use.

## 1. Introduction

Materials that simultaneously possess magnetic and electric ferroic orders belong to the multiferroic family and for the last two decades have attracted the attention of researchers in the field of functional oxides and materials science [1,2,3]. Ferrite-based materials, both single-phase [4,5,6] and composite [7,8,9], appear to be very interesting and practically significant. Materials in nanoform are very important at the moment, because they can have electronic properties that are more attractive than their bulk counterparts. Lately, many works have been devoted to obtaining and studying the properties of ferrites in nanoform [10,11,12,13,14]. In addition, magnetic materials are also of interest in nanoscale magnetic phase separation. It should be noted that functional oxides based on transition metal ions are promising materials for various technological applications as magnetic field sensors, working components in tunable microwave resonators, phase shifters, and other electronic devices and sensors [3,6,7,8,9,10].

One of the most promising materials in the area of multiferroics is bismuth orthoferrite (BiFeO_3_), which is characterized by transitions to magnetically ordered and ferroelectric state at temperatures well above room temperature, viz. T_N_ ~650 K and T_C_ ~1100 K, respectively [15,16]. It is known that chemical substitution of bismuth and iron ions allow to modify crystal structure of the compounds and thus change their physical properties, viz. magnetization, resistivity, electromechanical, and magnetoelectric parameters [17,18,19,20,21,22,23,24,25,26].

Chemical substitution by large rare earth ions (viz. La-Sm) settled in the A position of perovskite lattice leads to the following sequence of the structural transitions: polar rhombohedral-antipolar orthorhombic-nonpolar orthorhombic phase [19,27,28]. The compounds in the vicinity of the polar rhombohedral-antipolar orthorhombic phase boundary are characterized by rapid increase in piezoelectric coefficients as well as an increase in remnant magnetization. These phenomena have been associated with the metastable structural state ascribed to the compounds [19,29,30,31].

Substitution of bismuth ions with aliovalent alkali-earth ions Sr^2+^ and Ba^2+^ having ionic radii larger than that of Bi^3+^ ions leads to increase of the unit cell volume and causes structural transition from the polar rhombohedral phase to either the pseudo-cubic [32,33] or tetragonal phase [19,34]. Structural transformation occurs at the doping level of about 15–20%, regardless of the method used to prepare the compounds. Chemical substitution of Bi^3+^ by alkali-earth ions having a smaller ionic radius (e.g., Ca^2+^) causes more complicated structural transformations [29,30]. It should be noted that the threshold dopant concentration inducing the phase transitions to pseudo-cubic phase significantly depends on preparation conditions, oxygen content, and average grain size of the compounds. The mentioned factors hamper a determination of crystal structure evolution across the mentioned transitions and lead to ambiguous results in the literature [19,27,33].

Simultaneous doping at the A- and B-positions of the perovskite lattice allows to modify the sub-lattices responsible for polar and magnetic orders [35,36,37]. Thus, in compounds Bi_1−*x*_Ba*_x_*Fe_1−*x*_Ti*_x_*O_3_, an increase of the dopant content leads to structural transition from the polar rhombohedral phase to the polar tetragonal phase through the formation of an intermediate (pseudo)-cubic structure [8,37,38,39]. The compounds in vicinity of the rhombohedral-pseudocubic phase boundary are characterized by increased piezoelectric coefficient d_33_, reaching maximum value of about 250 pm/V in the compounds with 33% of BaTiO_3_ content. It should be noted that structural state of the solid solution with one-third of BaTiO_3_ content is characterized by the dominant pseudocubic phase with minor (~35%) fraction of the rhombohedral phase [37,38,39]. Moreover, our recent investigation involving the piezoresponse force microscopy measurements has revealed a polarizable state in compounds with *x* ~0.33, 0.35, while according to the XRD data, these compounds possess single phase cubic structure [37].

The available data describing the evolution of the crystal structure in the system Bi_1−*x*_Ba*_x_*Fe_1−*x*_Ti*_x_*O_3_ as well as the physical parameters declared in the different studies [37,40,41,42,43] have an ambiguous character and should be clarified. Our recent temperature-dependent structural measurements [38] performed for the compounds having dominant a rhombohedral phase within the mentioned phase boundary have revealed unexpected strengthening of the rhombohedral distortions at elevated temperatures. The most recent structural data obtained by the authors point to the non-monotonous character of the phase evolution from the polar rhombohedral to the cubic-like phase assuming a reinforcement of polar type distortion, which also affects piezoresponse in the compounds. Wide concentration range (~10%) of the phase coexistence area estimated for the compounds Bi_1−*x*_Ba*_x_*Fe_1−*x*_Ti*_x_*O_3_ facilitates careful and detailed analysis of the phase evolution driven by the dopant content and temperature. The current study is focused on structural aspects of the dopant and temperature-driven evolution of the structural state in the Bi_1−*x*_Ba*_x_*Fe_1−*x*_Ti*_x_*O_3_ compounds within the phase boundary region.

## 2. Materials and Methods

Polycrystalline compounds of Bi_1−*x*_Ba*_x_*Fe_1−*x*_Ti*_x_*O_3_ with 0.2 ≤ *x* ≤ 0.4 were prepared by a two-stage ceramic technique [38] using high-purity (≥99.5%) oxides Bi_2_O_3_, Fe_2_O_3_, TiO_2_, and carbonite BaCO_3_. The reagents taken in the following contents of the components *x* = 0.2 (20 mol.% of BaCO_3_ and TiO_2_), 0.25, 0.27, 0.3, 0.33, 0.35 and *x* = 0.4 were thoroughly mixed in a planetary ball mill (Retsch PM 200, Haan, Germany). Preliminary synthesis of the compounds pressed into tablets was performed at 900 °C. Final synthesis lasted for 20 h with temperature gradually increasing with the dopant content from 920 °C for the compound with *x* = 0.2 to *T* = 950 °C for the compounds with *x* = 0.4. After synthesis, the samples were quickly (100 °C/h) cooled down to room temperature.

Phase purity of the compounds was attested by X-ray diffraction (XRD) measurements recorded in the 2 Thetta range 20–80° with a step of 0.02° using a Bruker D8 diffractometer (Bruker Corporation, Billerica, MA, USA) with Cu-Kα radiation. The diffraction data were analyzed by the Rietveld method using the FullProf software (v2.5, ILL, Grenoble, France) [44]. Differential scanning calorimetry (DSC) and thermal analysis (DTA) were performed using a Mettler-Toledo GmbH (Mettler-Toledo GmbH, Columbus, OH, USA) instrument in argon flow. Scanning electron microscopy (SEM) images were taken for the morphology characterization with Hitachi SU-70 SEM (Hitachi Ltd., Tokyo, Japan). Piezoresponse force microscopy was done using a Ntegra Prima commercial scanning probe microscope (NT-MDT, Spectrum Instruments, Zelenograd, Moscow, Russia) equipped with external Zurich instruments HFLI lock-in amplifier and Scansens HA_NC tips (Scansens, Hamburg, Germany) with (stiffness 12 N/m, 30 nm nominal radius) under AC voltage with amplitude *V_ac_* = 6 V and frequency *f* = 21 kHz. Cantilever displacements was evaluated from vertical and lateral force calibrations [45]. Calibration of the probe tip displacements was made based on the quasi-static force-distance curves.

## 3. Results and Discussion

**Figure 1:**
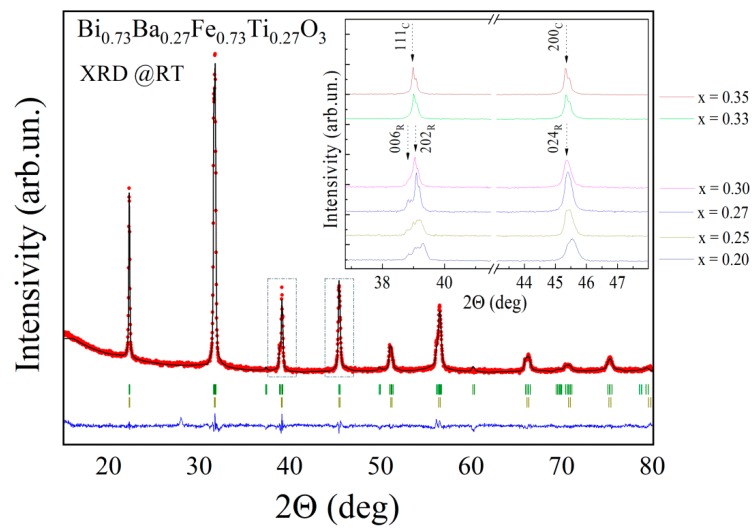
Room-temperature XRD pattern of compound Bi_0.73_Ba_0.27_Fe_0.73_Ti_0.27_O_3_. Observed and calculated profiles are marked by dots and solid line, respectively; the line below the pattern refers to the difference between the profiles. The upper row of the ticks denotes Bragg reflections ascribed to the rhombohedral phase, the second row—to the cubic phase. The inset shows concentration-driven evolution of the selected diffraction peaks.

**Figure 2:**
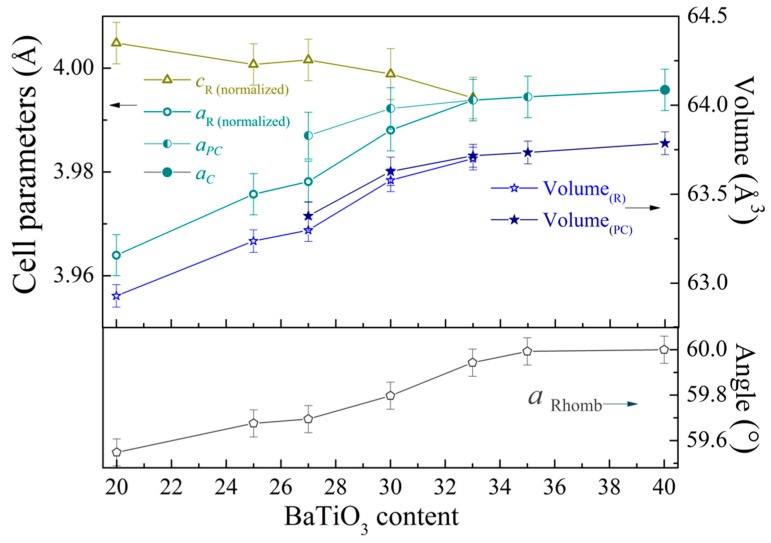
The dopant concentration driven evolution of the unit cell parameters, unit cell volume of the rhombohedral and pseudo-cubic phases, and the rhombohedral angle α_R_ calculated for compounds with 0.2 ≤ *x* ≤ 0.4. The a_R_- and c_R_- parameters are presented in normalized form, viz. calculated as a_R(norm)_ = a_R_/√2·and c_R(norm)_ = c_R_/2·√3, where a_R_- and c_R_- are parameters of the rhombohedral lattice in hexagonal settings.

The results of the diffraction measurements testify that compound with *x* = 0.2 is characterized by the single-phase structural state with rhombohedral distortion (space group *R3c*); the magnitude of the structural distortion was quite low compared to the initial compound BiFeO_3_ [18,21]. The character of the structural distortion was confirmed by negligible intensity of the specific reflection 113_R_ and weak splitting of the reflections 202_R_ and 006_R_ (Figure 1). Increase in Ba and Ti content leads to an increase in unit cell volume accompanied with a gradual reduction of rhombohedral distortion. The angle α_R_ describing rhombohedral distortion gradually increases from 59.55° for the compound with *x* = 0.2 up to 59.98° for the compound with *x* = 0.35, where rhombohedral distortion is hardly detectable (Figure 2). Modification of the unit cell parameters *a-* and *c-* shows a different character on chemical doping. The *a-* parameter gradually increases with the dopant content, while *c-* parameter remains nearly constant and start to decrease in the compounds with *x* ≥ 0.27, so the c/a ratio decreases down to unity in the compound with *x* = 0.35 (Figure 2).

**Figure 3:**
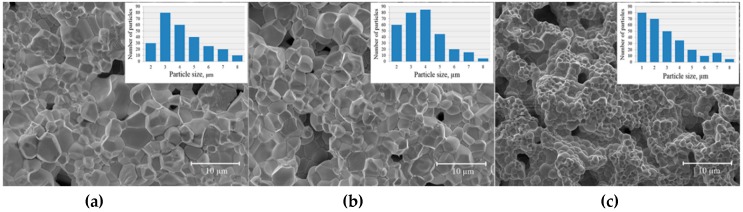
SEM images of the compounds Bi_1−*x*_Ba*_x_*Fe_1−*x*_Ti*_x_*O_3_ with *x* = 0.27 (**a**), 0.3 (**b**), and 0.35 (**c**) (left to right). The insets show the distribution of crystal grain size.

While the XRD patterns of the compounds with 0.2 < *x* < 0.3 can be satisfactorily refined using a single phase model with rhombohedral distortion, adding to the model the second cubic phase (space group *Pm-3m*) notably improves the reliability factors of the refinement. Slight asymmetry of the reflections ascribed to the rhombohedral phase confirms the presence of the secondary phase with larger unit cell parameters as compared to the rhombohedral phase (Figure 1, inset). It should be noted that the XRD patterns of the compounds with 0.2 ≤ *x* ≤ 0.3 are characterized by notable broadening of the X-ray reflections, as compared to other compounds within the phase boundary region (Figure 1, inset). An observed widening of the peaks is in accordance with a reduction in the average size of crystallines, as confirmed by SEM images (Figure 3). Micrometer size of the crystallines as well as a two-phase structural state of the compounds significantly reduces accuracy of the calculations using the Scherrer equation [46,47]. According to the SEM data, average size of the particles gradually decreases with dopant content (Figure 3). It should be noted that significant broadening of the X-ray reflections is observed only for compounds with *x* ≤ 0.3, unlike the compounds with higher dopant content (Figure 1, inset). The obtained results of XRD and SEM experiments for the compounds with *x* ≤ 0.3 can be explained in the model, which assumes the presence of the two different structural phases coexisting at the nanoscale level.

A reduction in the average crystalline size causes an increase of grain boundary volume fraction, which can hamper estimation of the ratio of different structural phases based on the diffraction data [48]. The piezoresponse force microscopy (PFM) results presented below for two-phase compounds confirm the mentioned issue, while the tendency of the phase evolution can be distinctly clarified based on the diffraction results. Based on the obtained results, one can conclude that the concentration driven structural transition from the polar rhombohedral to cubic-like phase occurs through coexisting phases having nanoscale average size and this effect is mostly pronounced in compounds with 0.2 ≤ *x* ≤ 0.3. Further increase in the dopant content is associated with an increase in the fraction of the cubic-like phase and its average size up to microscopic level, as confirmed by narrow X-ray reflections. The EDS analysis shows high chemical homogeneity of the compounds under study. The difference in the content of Bi and Ba ions calculated for different places within one grain as well as for the data obtained for different grains of the compounds is about 1%. The obtained results could not allow to distinguish crystallines ascribed to either rhombohedral or cubic-like phase, thus confirming the nanoscale character of different structural phases coexistent in the crystallines; this phenomenon is most pronounced in compounds with 0.2 ≤ *x* ≤ 0.3.

According to diffraction data, the phase coexistence region lasts till concentration *x* ~0.35 and the compounds with *x* ≥ 0.35 can be considered as a single phase having cubic structure. Local scale measurements performed by piezoelectric force microscopy (PFM) provide complementary information about the structural state of the compounds with mixed rhombohedral and pseudo-cubic phases.

**Figure 4:**
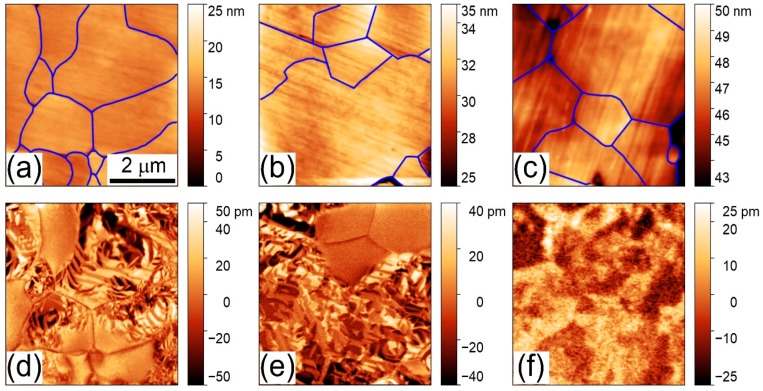
Topagraphy (**a**–**c**, crystallines are marked by solid lines) and out-of-plane PFM images (**d**–**f**) of the compounds Bi_1−*x*_Ba*_x_*Fe_1−*x*_Ti*_x_*O_3_ with *x* = 0.20 (**a**,**d**), 0.25 (**b**,**e**), 0.27 (**c**,**f**).

The results of the local piezoelectric measurements performed for the compounds Bi_1−*x*_Ba*_x_*Fe_1−*x*_Ti*_x_*O_3_ with 0.2 ≤ *x* ≤ 0.35 demonstrate a decrease of piezoresponse signal with dopant content. According to the XRD data, the dopant content leads to a reduction in “tetragonality” (e.g., c_R(norm)_/a_R(norm)_ ratio, which allows to estimate elongation of the lattice along c_R_ axis and thus evaluate changes in polarization value [45,46,47]) of the unit cell (Figure 1) and decrease in off-center displacement of Bi(Ba) ions, which are mainly responsible for polar properties of the compounds. Gradual reduction of the piezoelectric signal observed by PFM measurements is in accordance with continuing modification of the crystal structure of the compounds. The PFM data also show a distribution of the polar active phase throughout a surface of the compounds, and the obtained data allowed to itemize the polar active and the non-polar phases within separate crystallines (Figure 4). Figure 4 shows that the polar regions contain a clearly distinguished domain structure, while the non-polar grains are characterized by the absence of piezoelectric signal. The PFM data obtained for the compounds with *x* = 0.27 do not show a clearly distinguished domain structure (Figure 4), which can be caused by weak piezo signals and high mixing of the structural phases resembling relaxor-like behavior.

Based on the structural data and complementary results obtained by the PFM method, one can ascribe polar active regions to the rhombohedral phase, while the regions without distinct PFM contrast can be attributed to the cubic-like phase considered from the XRD data. It should be noted that PFM data were able to distinguish low intensity piezoelectric signal in the compounds having single phase cubic-like structure (e.g., *x* = 0.35) determined by the diffraction experiments. The observed phenomena can be explained by assuming the presence of polar distortion of the unit cell specific for the mentioned compounds, while the magnitude of the structural distortion was too low to be detected by conventional diffraction methods.

**Figure 5:**
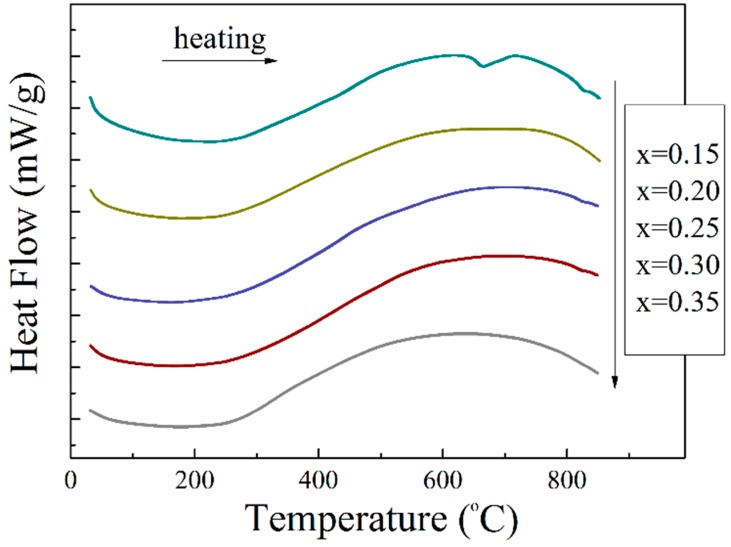
The dependency of heat transfer curves (DSC) obtained for the compounds with 0.15 ≤ *x* ≤ 0.35.

Temperature-dependent structural measurements allowed to itemize complex structural state ascribed to the compounds within the phase boundary region. Evolution of the crystal structure as a function of temperature was analyzed based on X-ray diffraction data as well as the results of DSC/DTA measurements. The compounds having a two-phase structural state at room temperature show gradual transition into single phase state characterized by a cubic structure. Temperature of the structural transformation into the cubic phase decreases with dopant content from ~ 650 °C for the compound with *x* = 0.2 down to ~350 °C for the compound with *x* = 0.35, which is in accordance with previously published data [37]. It should be noted that the evolution of mixed structural state into single phase cubic structure is characterized by small enthalpy associated with the transition and conventional DSC measurements performed for the compounds could not reveal this structural transition, unlike the structural data detecting this phenomenon (Figure 5). In contrast, the structural transition from the rhombohedral phase to the cubic phase observed in the compound with *x* = 0.15 can be easily detected by DSC measurements as well as the diffraction experiments [38]. The evolution of the mixed structural state into the single phase state confirms the intrinsic character of the phase separation observed for the compounds with 0.2 ≤ *x* ≤ 0.35, which is associated with high chemical homogeneity of the compounds. The obtained structural data allowed to reveal notable peculiarities of the temperature-driven structural transition, which was not declared in previous studies on the BiFeO_3_–BaTiO_3_ system [37,40].

**Figure 6:**
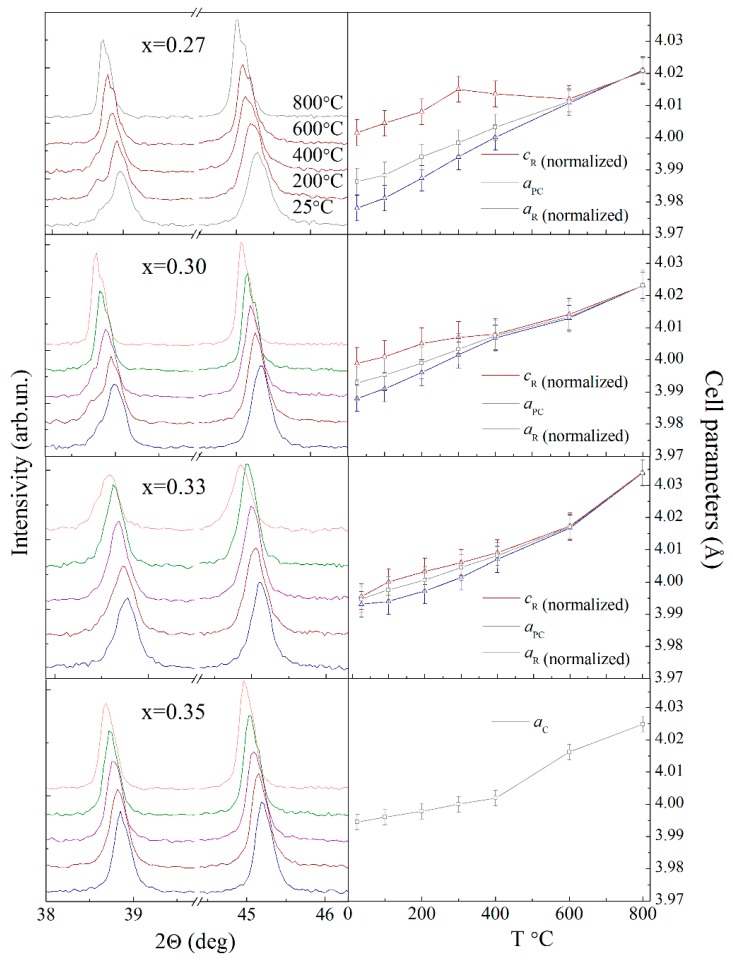
Temperature evolution of the diffraction peaks 006|202_R_ and 024_R_ and the unit cell parameters calculated for the compounds with 0.27 ≤ *x* ≤ 0.35. Temperature evolution of the diffraction peaks 006|202_R_ (located at ~38.9 deg.) and 024_R_ (located at ~45.2 deg.) and the unit cell parameters calculated for the compounds with 0.27 ≤ *x* ≤ 0.35.

The X-ray diffraction data obtained for the compounds under study testify an increase in unit cell parameters with temperature increase, while the *a-* and *c-* parameters of the rhombohedral lattice change in different ways. The evolution of the *a-* parameter shows nearly linear increase with temperature, while an increase in the magnitude of the *c-* parameter demonstrates a non-monotonous character. The calculated structural data testify of gradual increase in the magnitude of the *c-* parameter, followed by a rapid decrease after certain temperature, and the temperature of this modification decreases with dopant content (Figure 6). It should be noted that the observed modification in the unit cell parameters occurs at temperatures notably lower than the temperature of structural transition to the cubic phase. Unusual behavior of the unit cell parameters can be directly evidenced by an evolution of specific diffraction peaks. Careful analysis of the XRD patterns reveals that splitting of the rhombohedral reflections 006 and 202 becomes more pronounced at elevated temperatures (Figure 6).

For compound with *x* = 0.27, the noted effect is observed in the temperature range of ~130–240 °C and the mentioned temperature range declines and shrinks with dopant content down to nearly room temperature for the compound with *x* = 0.33 (Figure 6). A strengthening of the rhombohedral distortion observed at elevated temperatures for the compounds within the phase boundary region resembles a modification of the crystal structure driven by dopant concentration. An increase in the fraction of the cubic phase driven by either dopant content or temperature leads to competing structural distortions. Splitting of the rhombohedral reflections 006_R_ and 202_R_ is associated with elongation of the unit cell and thus with polar distortion of the lattice mainly caused by off-center displacement of the Bi(Ba) ions.

**Figure 7:**
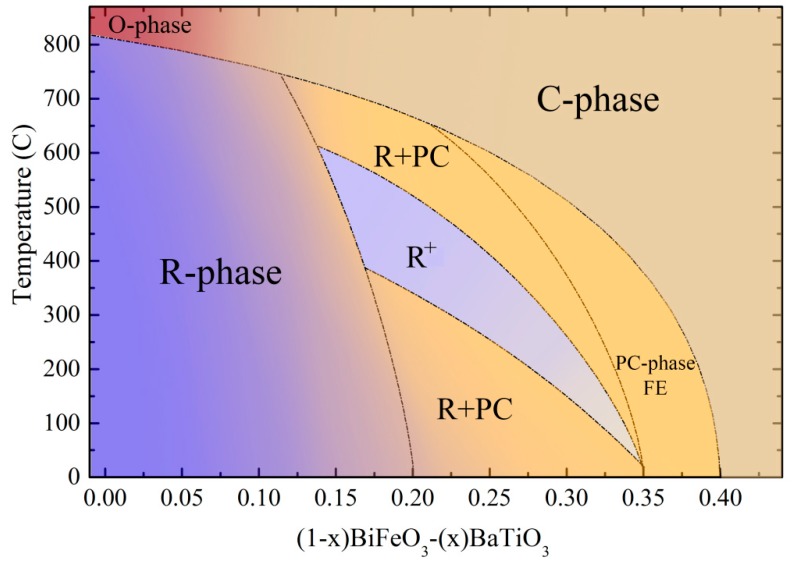
Structural phase diagram of the Bi_1−*x*_Ba*_x_*Fe_1−*x*_Ti*_x_*O_3_ compounds with 0 < *x* < 0.5, the phase boundaries between single phase and phase coexistent states are drawn by the dot-dashed lines; the region ascribed to strengthening of the rhombohedral distortion is marked by as R^+^.

Based on the obtained diffraction data, the results of DSC/DTA and PFM measurements performed for Bi_1−*x*_Ba*_x_*Fe_1−*x*_Ti*_x_*O_3_ solid solutions, as well as our previous data (reported in [38]), the temperature-composition phase diagram was clarified (Figure 7).

The phase diagram represents the concentration and temperature regions of the single phase and phase coexistent states. The concentration-driven transition from the polar rhombohedral phase to the cubic phase for the compounds at room temperature is located in the dopant content range 0.20 ≤ *x* ≤ 0.35. One should note that this transition occurs through the formation of the pseudo-cubic phase having insignificant polar distortion undetectable by the diffraction measurements. The compounds having dominant rhombohedral phase in the phase boundary region at room temperature are characterized by temperature-driven transition into the single phase cubic structure, wherein this structural transformation is accompanied by a strengthening of the rhombohedral distortion at elevated temperatures, thus showing the non-monotonous character of the phase transition.

## 4. Conclusions

Based on the obtained results, we conclude that the concentration-driven structural transition from the rhombohedral polar phase to the cubic phase extends in the range 0.20 ≤ *x* ≤ 0.35 and occurs through the formation of a polar active pseudocubic phase. The phase transition is characterized by a formation of coexistent structural phases of nanoscale size. The crystal structure of the compounds having a dominant pseudo cubic phase is considered to possess polar displacements of ions that are too slight to be detected by conventional diffraction methods. The two-phase compounds are characterized by strengthening of the rhombohedral distortion that occurred at elevated temperatures. Observed strengthening of the rhombohedral distortion reflects a non-monotonous change in the ratio of the coexistent phases within the phase transition and can be caused by competing trends associated with rotation of oxygen octahedra and dipole interactions changing with dopant concentration increase. The obtained structural data provide novel information on the mechanism of the structural transitions occurring in the BiFeO_3_–BaTiO_3_ system, which is important for development of new functional materials based on complex oxide systems.
